# Photo Quiz: Unexpected pathogens in the Gram staining of a blood culture smear

**DOI:** 10.1128/jcm.01925-24

**Published:** 2025-05-14

**Authors:** Jan Esse, Julius Sommer, Johannes Träger, Richard Strauß, Julia Fürst, Giuseppe Valenza, Christian Bogdan, Jürgen Held

**Affiliations:** 1 Mikrobiologisches Institut - Klinische Mikrobiologie, Immunologie und Hygiene, Universitätsklinikum Erlangen und Friedrich-Alexander-Universität (FAU) Erlangen-Nürnberg 160504 , Erlangen, Germany; 2 Medizinische Klinik 1, Universitätsklinikum Erlangen und FAU Erlangen-Nürnberg 72175, Erlangen, Germany; 3 FAU Profile Center Immunomedicine, FAU Erlangen-Nürnberg 9171, Erlangen, Germany; Mayo Clinic Minnesota, Rochester, Minnesota, USA

## PHOTO QUIZ 

A 57-year-old woman with known arterial hypertension consulted her general practitioner because of malaise and fever of undetermined character and duration. She was empirically treated with oral amoxicillin/clavulanic acid. One week later, she was admitted to the hospital and transferred to the intensive care unit due to impaired consciousness, shock (lactate 7.9 mmol/L), fever, leukocytosis (leukocytes 23.22 /nL), elevated inflammatory markers (C-reactive protein 231.9 mg/L, procalcitonin 150.4 µg/L), anemia (hemoglobin 86 g/L), thrombocytopenia (platelets 128 /nL), acute renal dysfunction (serum creatinine 41.6 mg/L), and acute liver failure. The patient was intubated, and mechanical ventilation and continuous renal replacement were started. Due to continuous veno-venous hemodialysis, also aiming for normothermic temperature management, no statements can be made about the course of the fever while being treated in the intensive care unit. Computed tomography of the head did not show any pathological findings. Blood cultures were sampled as part of standard microbiology diagnostics. After 3 h and 13 min of incubation, the aerobic blood culture bottle signaled growth (BD BACTEC Plus Aerobic/F blood culture bottle; BACTEC FX blood culture system, Becton Dickinson GmbH, Germany), and the Gram stain of a blood culture smear revealed numerous circular pathogens (Fig. 1A). Despite prompt treatment and maximal intensive care, multi-organ failure progressed, and the patient died within 1 day.

What is your diagnosis?

## ANSWER TO PHOTO QUIZ

The patient was diagnosed with malaria tropica. Subsequent Giemsa staining of the blood culture identified the circular pathogens within the erythrocytes as *Plasmodium falciparum* trophozoites (Fig. 1B). The BinaxNOW Malaria test (Abbott Diagnostics, USA) showed a positive reaction for the *Plasmodium falciparum*-specific histidine-rich protein II (HRPII) and the aldolase for pan-*Plasmodium* detection. Molecular analysis was not performed. A Giemsa-stained peripheral blood smear showed the typical ring forms and non-enlarged erythrocytes with up to five trophozoites per erythrocyte, confirming the diagnosis. The parasitemia was very high with 25% of erythrocytes being infected. Bacterial subcultures of the positive blood culture bottle and control blood cultures, collected after intravenous artesunate administration, remained negative and ruled out bacterial co-infection. The Sysmex XN-20 haematology analyzer (Sysmex Deutschland GmbH, Germany) used for automated blood cell counting flagged the patient’s blood sample for abnormal red blood cells, but not specifically for parasitic red blood cells as seen in a previous case report (1). The patient’s relatives confirmed that she had returned from a vacation in Tanzania and Zanzibar 3 weeks prior and had not taken any drugs for malaria prophylaxis. Despite immediate treatment with intravenous artesunate, the patient died a few hours after admission.

So far, only a few cases of positive blood cultures due to *Plasmodium falciparum* have been reported in the literature, with parasitemias between 1.8% and 19% (2–7). In our case, the very high parasitemia of 25% and the very short time to positivity of the blood culture were striking. When considering all reported cases, there was a trend towards a shorter time to positivity with higher parasitemia (Pearson’s correlation coefficient r = −0.633, *P* = 0.092, *n* = 8). Asexual blood stages of *Plasmodium falciparum* generate carbon dioxide via tricarboxylic acid metabolism (8) in amounts that can be sufficient to be detected by an automated blood culture system. A review of the literature revealed no report of positive blood cultures due to other protozoa.

In conclusion, positive blood cultures are not only caused by bacteria and fungi but also by *Plasmodium falciparum* and possibly other *Plasmodium* species. Our case highlights the importance of taking a complete medical history, including traveling and other risks for less common infectious diseases.
